# Interactions between antidiabetic drugs and herbs: an overview of mechanisms of action and clinical implications

**DOI:** 10.1186/s13098-017-0254-9

**Published:** 2017-07-26

**Authors:** Ramesh C. Gupta, Dennis Chang, Srinivas Nammi, Alan Bensoussan, Kellie Bilinski, Basil D. Roufogalis

**Affiliations:** 1 NICM, Western Sydney University, Locked Bag 1797, Penrith, NSW 1797 Australia; 2 Department of Agricultural Chemistry and Soil Science, School of Agricultural Sciences and Rural Development, Nagaland University, Medziphema, 797 106 India; 3 School of Science and Health, Western Sydney University, Locked Bag 1797, Penrith, NSW 1797 Australia; 4 Discipline of Pharmacology, School of Medical Sciences, Sydney Medical School, The University of Sydney, Sydney, NSW 2006 Australia

**Keywords:** Herb–drug interactions, Antidiabetic drugs, Antidiabetic herbs, Pharmacokinetic interaction, Pharmacodynamics interaction, Synergism

## Abstract

Diabetes is a complex condition with a variety of causes and pathophysiologies. The current single target approach has not provided ideal clinical outcomes for the treatment of the disease and its complications. Herbal medicine has been used for the management of various diseases such as diabetes over centuries. Many diabetic patients are known to use herbal medicines with antidiabetic properties in addition to their mainstream treatments, which may present both a benefit as well as potential risk to effective management of their disease. In this review we evaluate the clinical and experimental literature on herb–drug interactions in the treatment of diabetes. Pharmacokinetic and pharmacodynamic interactions between drugs and herbs are discussed, and some commonly used herbs which can interact with antidiabetic drugs summarised. Herb–drug interactions can be a double-edged sword presenting both risks (adverse drug events) and benefits (through enhancement). There is a general lack of data on herb–drug interactions. As such, more rigorous scientific research is urgently needed to guide clinical practice as well as to safeguard the wellbeing of diabetes patients.

## Background

Diabetes mellitus refers to a group of chronic metabolic diseases which are generally characterised by hyperglycaemia, which eventually leads to damage of multiple body systems. There are two types of diabetes, type 1 (T1DM) and type 2 (T2DM) diabetes mellitus. T1DM is referred as insulin-dependent diabetes mellitus (IDDM) and is caused by the impaired production of insulin. T2DM, however, is commonly associated with the inability of cells to respond to insulin (insulin resistance) and hence referred as non-insulin dependent diabetes mellitus (NIDDM).

The prevalence of diabetes has been increasing globally. In 2015, an estimated 415 million adults were living with diabetes, and this number is projected to increase to 642 million by 2040 [1]. Over 70% of those with T2DM live in developing countries, and this proportion is increasing annually [2]. In Australia, diabetes is among the top 10 leading causes of death and was responsible for 3% of all Australian deaths in 2011, whereby the most common cause of diabetes related death was coronary heart disease, accounting for 64% of deaths from diabetes [3].

Evidence suggests that lifestyle changes such as exercise, diet and other nonpharmacological interventions can delay and even prevent the development of T2DM. However, compliance to these interventions is low; with only about 50% of those with chronic illnesses have been shown to adhere to recommended lifestyle interventions [4]. Many antidiabetic pharmaceutical drugs are available, but the increase in the incidence of T2DM, especially in developing countries, together with adverse events associated with these drugs, has highlighted the need for more effective, safer and less costly management approaches.

The global use of complementary and alternative medicine (CAM) for the management of diseases such as diabetes has rapidly increased over the last decade. It is reported that up to 72.8% of people with diabetes used herbal medicine, dietary supplements and other CAM therapies [5]. Furthermore, research indicates that most people who use CAM therapies do so in addition to, rather than in place of, conventional medicine [6]. A large number of medicinal plants are believed to possess antidiabetic properties and have been utilised to manage diabetes [7–9]. However the concurrent use of antidiabetic herbs and pharmaceutical medicines has raised safety concerns. Unlike pharmaceutical medicines, where the ingredients are well defined and characterised, herbal medicine contains multiple bioactive components for which there is a lack of understanding of how these components interact with each other and with pharmaceutical medicines when taken in combination.

Although many studies regarding herb–drug interactions emphasise the potential harmful effects of such interactions, the possibility of herbal components beneficially enhancing or facilitating the action of antidiabetic pharmaceutical agents (or vice versa) may also exist. Positive interactions between herbs and drugs may lead to enhanced effectiveness of the antidiabetic agents through additive or synergistic actions. This review aims to provide an overview of the studies investigating interactions between antidiabetic herbs and conventional medicine, identifying of both negative and positive aspects of these interactions.

## Herb–drug interaction and its mechanisms of action

**Fig. 1:**
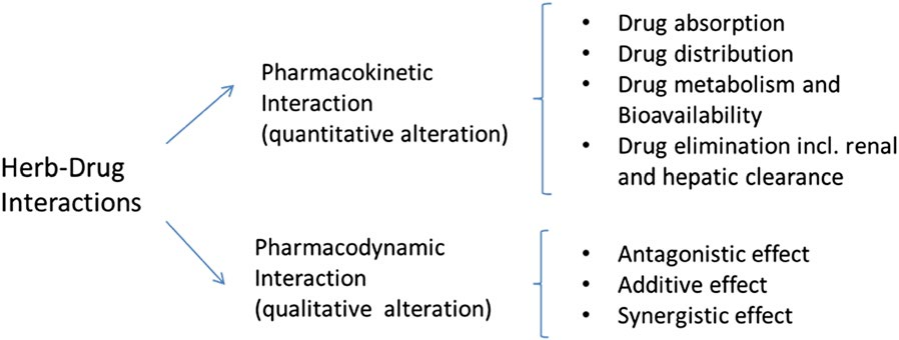
Mechanisms of action of herb–drug interactions

Two (or more) drugs when administered together have the potential to cause chemical or pharmacological interactions. Such interactions may alter the effect of either agent, leading to decreased or increased effectiveness or severity of adverse effects. The outcomes are dependent on many chemical and pharmacological factors, such as the physicochemical nature of the drugs in use and how they affect each other pharmacokinetically and pharmacodynamically (Fig. 1). Although, the mechanisms of interactions between herbs and drugs are similar, they are more complex in nature when several compounds are involved. Herb–drug interactions (HDI) may affect clinical safety and efficacy via additive/synergistic or antagonistic interactions among the herbal components and drug molecules. Whilst negative or harmful interactions tend to receive more attention due to safety considerations, additive/synergistic effects induced by HDIs may result in an enhancement of desired pharmacological effects. For example, the blood glucose lowering effect of antidiabetic drugs has been shown to be increased by agrimony [10].

A number of mechanisms may be associated with pharmacokinetic HDIs including quantitative alterations in renal clearance [11, 12], bioavailability [13], drug distribution [14, 15], absorption [16–18], and elimination processes [19]. Hepatic metabolic enzyme systems, particularly the cytochrome P450 (CYP450) isoenzyme family, remain a common pathway for pharmacokinetic HDIs. Many anti-diabetic drugs are substrates of CYP450 isoenzymes, e.g. pioglitazone, repaglinide and rosiglitazone for CYP2C8, glibenclamide, glimepiride, glipizide, nateglinide and rosiglitazone for CYP2C9, proguanil for CYP2C19, and pioglitazone and repaginate for CYP3A4 [20–22]. A large number of herbs have also been suggested to affect the CYP450 system. For example, St John’s wort inhibits CYP2C and CYP3A and ginkgo inhibits CYP3A4, CYP2C9 and CYP2C19 [23].

Pharmacodynamic HDIs can modify the drug/herb actions in a qualitative manner through effects on various organs, receptor sites or enzymes. Such interactions can result in antagonistic, additive or synergistic effects. For example, many herbal medicines possess antioxidant properties which could be beneficial for reducing oxidative stress, a key pathogenic factor of diabetes [24–26]. Several pharmaceutical agents effective in reducing diabetic mortalities (e.g., 3-hydroxy-3-methylglutaryl coenzyme A reductase inhibitors) have also been shown to have antioxidant activities [24]. When these herbs and drugs are used together, pharmacodynamic HDI (either additive/synergistic) may occur. Some of the known interactions between selected antidiabetic drug and antidiabetic herbs are discussed in “Common herb–drug interactions in diabetes” [24–26].

## Antidiabetic pharmaceutical and herbal interventions

### Common antidiabetic drugs

Several groups of pharmaceutical agents are currently used for the treatment of diabetes via different mechanisms, such as stimulation of the release of insulin (e.g., sulfonylureas), reduction of hepatic glucose output and enhancement of the peripheral uptake of glucose (e.g. biguanidines) [27–29]. Some of the commonly used antidiabetic drugs include biguanides, e.g., metformin (via acting directly to influence insulin resistance), peroxisome proliferator activated receptor (PPAR) activators, e.g., thiazolidindiones (via improving insulin resistance), vidagliptin and other related “gliptins” (via blocking DPP-4, an enzyme that degrades the incretin GLP-1) and α-glucosidase inhibitors, e.g. acarbose and miglitol (via delaying the digestion of complex carbohydrates). Other diabetic agents target pancreatic beta-cell receptors by binding to the sulfonylurea receptor subunit, blocking the K^+^-ATP channel to promote insulin release [30, 31]. Additionally, combination therapies (e.g. sulfonylureas with biguanides, thiazolidinedione with glucosidase inhibitors) are widely used to broaden therapeutic targets in order to improve efficacy and to minimise side effects.

### Herbs with antidiabetic properties

An increasing number of medicinal plants are being used to treat diabetes and its related conditions. The current NAPRALERT database lists over 1300 species of plants representing more than 750 genera within 190 families, covering lower plants such as algae and fungi to almost all types of higher plants. Many of these plants have been used ethno-pharmacologically in traditional medicine as antidiabetics, particularly for T2DM [32, 33]. Although many of these plants have been studied experimentally to validate their physiological activity, the chemical and pharmacological properties underpinning the anti-diabetic activity is less well studied. Nevertheless, a large number of potentially bio-active molecules have been isolated and identified, among which include complex carbohydrates, alkaloids, glycopeptides, terpenoids, peptides, amines, steroids, flavonoids, lipids, coumarins, sulphur compounds and inorganic ions [32].

Examples of common herbs and dietary supplements that have been used to treat diabetes include *Momordicacharantia*, *Trigonellafoenum*-*graceum*, *Gymnemasylvestre*, *Azadirachtaindica*, l-carnitine, vanadium, chromium and vitamin E. Proposed mechanisms’ of action underlying the antidiabetic effects of these compounds include direct effects on insulin secretion, activation of glycogenesis and hepatic glycolysis, adrenomimeticism, pancreatic beta cell potassium channel blocker activity, cAMP activation, and modulation of glucose absorption from the intestine [34–36].

## Common herb–drug interactions in diabetes

The co-administration of antidiabetic herbs and pharmaceutical agents may result in HDIs leading to enhanced effects (which may be desirable clinically), decreased pharmacological effects, or adverse drug events, such as hypoglycaemia. The following section provides a brief discussion of common antidiabetic herbs and their potential interactions with antidiabetic agents. Literature searches were conducted with PubMed and Google Scholar up to June 2017. The selection of medicinal plants for inclusion is based on their consistent use over long periods and on the strength of available data on effectiveness or adverse/synergistic effects.

### Aloe vera—*Aloe barbadensis*

Aloe vera is native to Africa and is one of the more than 400 species of the genus Aloe. The presumed major active components include carbohydrates (e.g., mannan, galactose-rich polysaccharides), and galacturonic acid [37]. Traditional literature reveals a wide range of clinical uses of this plant from cosmeceuticals through to immunity and organ care. In diabetes, aloe vera has been shown to significantly reduce blood glucose levels [38]. Several studies report potential interactions between aloe vera and antidiabetic drugs. Of note is its interaction with glibenclamide, a sulphonylurea which exerts its antidiabetic potential by inhibiting ATP sensitive potassium channels in pancreatic β cells, resulting in cell membrane depolarization and subsequent insulin release. The combination of aloe vera and antidiabetics has generally been shown to have an additive effect. For instance, aloe has been shown to produce a greater anti-hyperglycaemic effect, when compared to the sole therapy with glibenclamide, pioglitazone or repaglinide [39–41].

### Ginseng-*Panax ginseng and Panax quinquefolium*

Both *Panax ginseng and Panax quinquefolium*, two important members of the ginseng family, have been shown to possess antidiabetic properties affecting insulin dependent and insulin independent pathways [42–44]. The bioactive constituents responsible for ginseng’s antidiabetic actions are likely to be attributed to its ginsenosides [45, 46]. Although the precise active components responsible for this anti-diabetic action are unknown, studies with compound K (CK), a final metabolite of protopanaxadiol ginsenosides demonstrate that CK exhibits anti-hyperglycaemic effects through an insulin secreting action similar to metformin. The combined treatment of CK and metformin has been shown to elicit additive effects compared to individual components being used alone. Significant improvements were observed in plasma glucose and insulin levels, homeostasis model assessment-insulin resistance (HOMA-IR) and in haematoxylin and eosin-stained liver tissues [45, 46].

### Karela—*Momordica charantia*

Karela is also known as bitter melon due to its taste. A large number of chemical constituents are found in its juice, including sterols, glucoside mixtures and charantin polypeptides [47]. Karela is one of the few medicinal plants that has been subjected to extensive clinical studies in combination with common antidiabetics. Increased efficacy has been reported when used together with metformin, glymidine and glibenclamide. In one clinical trial, 400 mg of chloroform/benzene karela extract was combined with 50% of the full clinical doses of either metformin or glibenclamide in NIDDM patients. Results showed that the combined interventions elicited a greater hypoglycemic effect when compared to that of full doses of metformin or glibenclaminde alone, indicating a possible additive effect [48]. Similar results have also been obtained in animal studies whereby the combined treatments of karela fruit juice/extracts and metformin have been shown to produce greater hypoglycemic effects than either treatment alone in rat models of diabetes [49–51].

### Ginger—*Zingiber officinale*

Ginger has been widely used as spice as well as medicine for many years. Crude ginger contains up to 9% lipids or glycolipids and about 5–8% oleoresin. The pungent principles, accounting for 25% of the oleoresins, consist mainly of gingerols and related phenolic compounds [52]. Its aqueous extract is in use as an antidiabetic in many countries as part of traditional therapy. It is believed that the antidiabetic effect of ginger is derived from its antioxidant and anti-glycation properties, and its ability to express the glucose transporter Glut 4 [53]. In a study by Al-Omaria [54] in a rat model of streptozotocin (STZ)-induced diabetes, a concurrent treatment of ginger extract (25 or 50 mg/kg) and glibenclamide (5 mg/kg) significantly reduced non-fasting blood glucose level by 26 and 25%, respectively, compared to 7.9% reduction when glibenclamide was used alone [54]. In another study, a combination of ginger extract and a sub-optimal dose of glibenclamide (0.5 mg/kg) was found to exert effects similar to a full therapeutic dose of glibenclamide (1 mg/kg) in the STZ-induced diabetic model, highlighting the possibility of reduced side-effects of antidiabetics (due to the lower dose required) when used in combination with ginger extract. In addition, ginger has been shown to have renal protective effects when used with metformin [55, 56].

### Prickly pear cactus—Nopal

Prickly pear cactus (Nopal) athough native to Mexico, is now widely used worldwide as food and traditional medicine. Cacti are divided into several genera, including Opuntia (e.g., *Opuntiaaciculata*). Opuntia contains a range of phytochemicals in variable quantities, such as polyphenols, dietary minerals and betalains, as well as various compounds including gallic acid, vanillic acid and catechins [57]. Prickly pear seeds have been found to increase muscle and liver glycogen and reduce blood glucose level in STZ-induced diabetic rats, possibly through an insulin sensitizing effect [58]. One study showed a positive interaction between the combined effect of prickly pear cactus pad and glipizide and metformin in T2DM patients. In this study a hypoglycaemic reaction was observed, although the authors note that clinical studies are required to support combined therapy of this herb and known diabetic drugs [58].

### Sesame oil

Sesame oil is obtained from sesame seeds and is widely used in cooking and as a flavour enhancer. It is composed of the following fatty acids: linoleic acid (41% of total), oleic acid (39%), palmitic acid (8%), stearic acid (5%) plus small amounts of other fatty acids [59]. Sesame oil has several traditional medicinal properties and has been reported to possess antidiabetic properties [60]. In a landmark clinical study by Sankar et al. 62 patients (32 male, 28 female) with T2DM were divided in 3 groups receiving sesame oil (~35 g oil/day used in cooking or salad preparation) alone, gliberclamide, orsesame oil and gliberclamide combination [61]. The combination group showed a greater anti-hyperglycaemic effect with a 43% reduction of glycosylated haemoglobin and 36% reduction of blood glucose level when compared to those receiving sesame oil and glibenclamide monotherapy. Improvements were also observed in enzymatic and non-enzymatic antioxidant levels in patients treated with sesame oil alone or in combination with glibenclamide, suggesting that sesame oil has an additive/synergistic effect when co-administered with glibenclamide [61].

### Fenugreek—*Trigonellafoenum*-*graecum*

Fenugreek is commonly used as a spice in south Asia and is known for its hypoglycaemic and hypocholesterolemic properties [62]. The proximate composition of fenugreek (seeds, husk and cotyledons) contains saponin, protein and polyphenols [63]. Interactions of fenugreek with known antidiabetics have been evaluated in several chemically induced diabetic animal models. The combination of fenugreek (150 mg/kg) and metformin (100 mg/kg) produced a significant reduction in plasma glucose level (20.7%) in type 2 diabetes [64]. In a similar study, lipid peroxidation (LPO) induced by ferrous sulphate, hydrogen peroxide and carbon tetrachloride in liver were performed. The combination treatment with fenugreek seed extract and glibenclamide exhibited a greater inhibition of the hepatic LPO activities and a greater antioxidant activity compared to the individual components alone, highlighting a potential benefit of the combination treatment [64].

### Garlic—*Allium sativum*

Garlic is known for its spectrum of medicinal properties. It is composed of a large number of sulfur compounds, with suspected bioactive compounds called allyl thiosulfinates (mainly allicin) [65]. Garlic has been reported to possess antidiabetic properties. Several experimental and clinical studies have been conducted to assess the interaction between garlic and antidiabetic medicines. In a rat model, the effects of garlic on the pharmacokinetic profiles of metformin were investigated. It was found that garlic increased the peak plasma concentration (C_max_) and the area under the curve (AUC) of metformin, highlighting the need to adjust the metformin dosage when co-administered with garlic [66]. In another study combination therapy of garlic extract (50 or 100 mg/kg) and metformin over 28 days was tested in a rat model of streptozocin-induced diabetes. Garlic alone, as well as in combination with metformin, improved body weight, whilst the combination therapy was more effective in reducing blood glucose levels, highlighting that garlic extract potentiates the hypoglycaemic effect of metformin [67]. Potential beneficial effects of garlic juice in combination with metformin have been shown, where the combination attenuated tubular toxicity induced by gentamicin [68, 69]. In a clinical trial, 60 diabetic patients with fasting blood sugar levels above 126 mg/dl were randomly divided in two groups to receive garlic tablets (300 mg thrice daily) and metformin (500 mg twice daily), or placebo and metformin over 24 weeks. A significantly greater reduction in blood glucose level (3–12%) was found in the group with co-treatment of garlic and metformin when compared to that of the placebo and metformin group (0.59%), indicating an enhancement effect [70].

### Gymnema—*Gymnema sylvestre*

Gymnema is native to South India and its pharmacological properties are mainly attributed to triterpenoidic saponins [71]. This herb has been in use for diabetic treatment for almost two millennia [72]. The interaction of gymnema (100 and 500 mg/kg orally) with metformin (50 and 100 mg/kg has been studied in STZ-induced diabetic rats. The combined treatment was found to decrease the bioavailability of metformin and serum glucose level; the decrease in serum glucose however was not significantly greater than that of metformin itself, although histopathological analyses showed an increase in volume of pancreatic islet cells after combined therapy [73]. In an animal study using a chemically-induced diabetic rat model a decrease in plasma metformin concentration and increase in blood glucose levels were seen in animals treated with the combination of gymnema tea and metformin when compared to those receiving metformin alone, suggesting an antagonistic interaction between metformin and gymnema [74]. In a similar study of chemically-induced diabetic rats, a significant decrease in bioavailability of metformin was observed which was proportional to the dose of gymnema used. However, the combined treatment significantly reduced the blood glucose level compared to individual administration of metformin or gymnema [75]. These findings suggest further research in individuals with diabetes is required to determine the effect of the combination of gymnema tea and metformin on blood sugar levels.

### St John’s wort—*Hypericum perforatum*

Although St John’s wort (SJW) is a medicinal herb with well-established as an antidepressant, it has also been reported to possess antidiabetic properties. The main bioactive components of the herb are thought to be naphthodianthrones, hypericin and pseudohypericin along with the phloroglucinol derivative hyperforinand essential oils (mainly sesquiterpenes) [76]. In a clinical pharmacokinetic study, 20 healthy male participants received 1 g metformin twice a day for 1 week, with and without 21 days preceding concomitant therapy with SJW. SJW decreased the renal clearance of metformin but had no effects on other pharmacokinetic parameters. Nevertheless, SJW treatment improved glucose tolerance by enhancing insulin secretion independent of insulin sensitivity [77]. However, these results differ to that of a study in which pre-treatment with SJW had no effect on blood glucose lowering or the insulin elevating effect of repaglinide [78]. Further research is required to clarify these findings.

### Astragalus—*Radix astragali*

Astragalus is a frequently used traditional Chinese medicine for diabetes. The bioactive constituents of astragalus include polysaccharides, triterpenoids (astragalosides), isoflavones (including kumatakenin, calycosin and formononetin), glycosides and malonates [79]. In Chinese herbal medicine astragalus is commonly used as a key herb in antidiabetic formulations. The effect of astragalus on the pharmacokinetics of pioglitazone has been investigated in a number of clinical and preclinical studies. In healthy human subjects, treatment of astragalus extract significantly reduced the C_max_ and increased final velocity (V/F) of pioglitazone whereas an opposite effect (i.e. increased C_max_ and reduced V/F) was observed in those with T2DM, although the reasons for this disease-dependent effect were unclear [80]. In a study in rats, co-administration of astragalus decoction and pioglitzone did not appear to alter the pharmacokinetic profiles of pioglitzone [80].

### Scutellaria—*Scutellaria baicalesis*

Scutellaria is a medicinal plant which roots are used to prepare traditional medicines. Several chemical compounds have been isolated from the root of scutellaria including baicalein, baicalin, wogonin, norwogonin, oroxylin A and β-sitosterol [81]. The effect of combined administration of metformin (500 mg/kg) and the ethanolic extract of scutellaria (400 mg/kg) for 30 days was examined in a rat model of STZ-induced diabetes. Combination treatment resulted in elevated hepatic activity of antioxidant enzymes compared with metformin alone. Hepatic lipid peroxide concentration was significantly reduced by combination treatment, with a corresponding reduction of plasma and hepatic triglycerides and cholesterol levels. These results suggest that scutellaria enhances the antidiabetic action of metformin although further research in individuals with diabetes is required to confirm these findings.

### *Andrographis paniculata*

*Andrographis paniculata* is a herb commonly used by individuals with diabetes [82]. Potentially additive pharmacological effects are apparent with the use of the herb in combination with antidiabetic medications as the herb has been shown to lead to enhanced uptake of radioactive glucose in the isolated soleus muscle of STZ-diabetic rats in a concentration-dependent manner [83]. Although there are no studies examining interactions between *Andrographis paniculata* and antidiabetic drugs, *Andrographis paniculata* has been shown to inhibit CYP2C19 activity [84] for which the antidiabetic drugs such as glibenclamide, glimepiride, glipizide, nateglinide, rosiglitazone, pioglitazone, repaglinideare substrates, thereby suggesting that there is the potential adverse outcomes as a result of an increase in plasma concentrations of these medications and subsequent enhanced glucose lowering effect, although this theory remains to be confirmed.

### *Lycium*—Berberislyceum Royle

*Lycium* is commonly found in the Himalayan region of India and Pakistan and is traditionally used as a medicinal plant for diabetes. Its hypoglycaemic effects are believed to be due to its bioactive polysaccharides and antioxidants. Evidence supporting the interaction between *Lycium* and antidiabetics is experimental only. The effect of 4 weeks treatment with *Lycium* (10 mg/kg/d) on blood glucose was examined in rats with STZ-induced T2DM [85]. Blood glucose levels in *Lycium* treated rats decreased by 34.9% (P < 0.01) compared with controls. Findings such as these suggest that *Lycium* may have an additive effect when used in combination with conventional antidiabetics [86]. However, evidence supporting Lycium’s antidiabetic activity in humans and interaction with antidiabetic medications is essential to determine whether similar effects are observed in human studies.

### Cassia—*Cassia fistula* and *Cassia occidentalis*

Cassia is an ethnomidicinal plant that is widely used in Indian and Chinese medicine to treat diabetes. It has been proposed that the antioxidant and polyphenol content of *Cassia fistula* and flavonoid content of *Cassia occidentalis* contribute to their antihyperglycaemic properties [87, 88]. Normal and STZ-induced diabetic rats were administered with 0.45 g/kg *Cassia fistula* hexane extract exhibited comparable effects to that of glibenclamide [87]. Similarly, *Cassia occidentalis* has been shown to have significant antihyperglycaemic activity in normal and alloxan-indiced diabetic rats [88]. Cassia inhibits enzyme activities of CYP2C9 for which glibenclamide, glimepiride, glipizide, nateglinide, and rosiglitazone are substrates, and CYP3A4 for which pioglitazone and repaglinide are also substrates [89], suggesting there may be an additive effect of this herb with antidiabetic medications.

### Olive leaf extract

Olive tree (*Olea europaea* L.) leaves have been widely used in traditional remedies in European and Mediterranean countries. They have been used as extracts, herbal teas, and powder and contain several potentially bioactive compounds that may have antioxidant, antihypertensive, antiatherogenic, anti-inflammatory, hypoglycemic, and hypocholesterolemic properties. Olive leaf polyphenols, in particular oleuropein aglhydroxytyrosoycone and its main metabolite, hydroxytyrosol, are considered the primary compounds responsible for these effects [90].

A number of experiments in cell and animal models and clinical trials have shown a beneficial effect of olive leaf extract in type 2 diabetes. One clinical trial involving 79 individuals with type 2 diabetes showed a significant reduction in HbA1c levels in those treated with olive leaf extract for 14 weeks (8.0 ± 1.5% vs. 8.9 ± 2.25%, P = 0.037) [91]. Compared with placebo, olive leaf extract treatment was also associated with a significant decrease in fasting insulin levels (11.3 ± 4.5 vs. 13.7 ± 4.1, P = 0.01). Approximately 90% of participants were treated by oral therapy for T2DM although the authors did not compare the effects of olive leaf extract between the two groups, and thus further research is required to determine whether there was an interaction between the olive leaf extract and oral hypoglycaemic medication.

Suggested mechanisms include the effect of olive polyphenols in preventing amylin aggregation in amyloid in pancreatic β-cells in the pancreas which impairs insulin-secreting cells [92].

## General discussions and conclusion

Based on the results presented above, it is clear that numerous herbal medicines, when taken in conjunction with antidiabetic pharmaceutical agents, could potentially alter their pharmacokinetic and/or pharmacodynamic properties. These interactions are complex given the large number of pathophysiological/pharmacological targets associated with the disease and the multicomponent properties of herbal medicine. The batch-to-batch variation in chemical composition of herbal medicine is also likely to impact on the nature of the interactions, making them unpredictable (Table 1).

**Table 1:** Herb–antidiabetic drug co-administration studies

Herb	Co-administered anti-diabetic drug	Experimental/clinical study	Observation	References
Aloe vera	Glibenclamide	Clinical	Additive effect on blood glucose lowering	[39, 40]
Andrographis paniculata	NA	Experimental	Antihyperglycaemic effect Inhibits CYP2C19 activity	[83, 84]
Cassia	Glibenclamide	Experimental	Comparable effect to glibenclamide	[87]
Ginseng (Ginsenoside CK)	Metformin	Experimental	Combined treatment with CK—ginsenoside and metformin has shown enhanced effect compared to individual compounds. Significant improvements were observed in plasma glucose and insulin levels	[45]
Karela-Bitter melon (*Momordicacharantia)*	Metformin	Clinical	Significant decrease in serum glucose was observed in combination of fruit juice extract at half the normal dose of metformin	[48]
	Glibenclamide	Clinical	Significant decrease in serum glucose was observed in combination of fruit juice extract at half normal dose of glibenclamide	[48]
	Metformin	Experimental	Fruit juice showed significant hypoglycemic effect in combination in normal, STZ- and alloxan-diabetic rats	[49–51]
Ginger (*Zingiber officinale)*	Glibenclamide	Experimental	Combination with ginger extract reduces blood glucose level greater than glibenclamide alone	[54]
			A sub-optimal dose of glibenclamide in combination with herb extract showed similar effects as a full therapeutic dose of glibenclamide	
	Metformin	Experimental	Ginger reduces hyperglycaemia and improved renal dysfunction in diabetic rats at reduced metformin dose. Combination of metformin and ginger juice ameliorates gentamicin nephrotoxicity	[55, 56, 117]
Lycium-*Berberislyceum royle*	Antidiabetics	Experimental	Significant reduction in glucose	[85]
Prickly pear cactus (Nopal)	Glipizide	Clinical	Hypoglycaemic adverse reaction with combination	[58]
	Metformin			
Sesame oil	Glibenclamide	Clinical	Improved anti-hyperglycaemic effect in combination	[61]
Fenugreek	Metformin	Experimental	Significant reduction in plasma glucose level	[64]
	Glibenclamide	Experimental	Seed extract and glibenclamide inhibited induced hepatic lipid peroxidation and exhibited higher antioxidant activity	[64]
Garlic	Metformin	Experimental	Herb is capable of affecting the pharmacokinetics of metformin resulting in reduced blood glucose level	[66]
		Experimental	Combination therapy has better reducing effect on blood glucose level	[67]
			Garlic with metformin in combination attenuates drug induced tubular toxicity	
		Experimental	Significant decrease in blood glucose level	[68, 69]
Gymnema	Metformin	Experimental	Decrease in bioavailability of metformin when given in combination with herbal tea; the combination did not decrease the serum glucose level compared to metformin alone	[73]
		Experimental	*Gymnema sylvestre* orally in chemically induced diabetic rats causes decreases in bioavailability of metformin and increase in blood glucose- therefore negative interaction observed	[74]
		Experimental	Beneficial pharmacodynamic effects on blood glucose reduction by combination compared to individual metformin; but reduced metformin bioavailability	[75]
St. John’s wort	Metformin	Clinical	Decreased renal clearance of metformin but no other pharmacokinetic effects. However SJW decreased the area under glucose concentration-time curve. Improved glucose tolerance by enhancing insulin secretion independently of insulin sensitivity in male subjects taking metformin	[77]
	Repaglinide	Clinical	No effect on blood glucose lowering and insulin elevating effects of repaglinide. No significant effect on pharmacokinetics and pharmacodynamics of repaglinide	[78]
Radix astragali	Pioglitazone	Experimental	Co-administration did not affect pharmacokinetics of pioglitazone	[80]
Scutellaria	Metformin	Experimental	Significant elevations of plasma and pancreatic levels and reduction of plasma and hepatic levels of triglycerides and cholesterol	[118]
			Herb enhanced the antidiabetic action of metformin	

In this review we have found that interactions of antidiabetic drugs and herbs may result in antagonistic or enhancement effects. The enhancement of glucose lowering has the possibility of causing hypoglycaemia, hence monitoring of potentially adverse effects is required and hence it is recommended that people with diabetes closely monitor their blood glucose levels when combining the two compounds. Although the vast majority of available evidence suggests that herbal medicines are relatively safe one case report showed that a patient with T2DM who was treated with the combination of Metformin and Repaglinide experienced hypoglycaemia [93], suggesting that patients and clinicians should indeed be alert to this possibility. Further research is required to examine the potential for hypoglycaemia in patients who are concurrently administered antidiabetic drugs.

Despite the potential for adverse effects, the combination of these herbs and antidiabetic medications has been more commonly shown to have positive clinical implications as it could lead to enhanced antidiabetic effects, potentially enabling a reduction in dose of antidiabetic agents, thereby minimising their side effects. In contrast, antagonism may lead to harmful effects and therefore warrant a cautionary warning or contraindication for the combination. Although not discussed in this review, antidiabetic herbs may also interact with other (non-diabetic) medicines when taken concurrently [94]. These considerations indicate that caution should always be exercised when herbal medicines are combined with pharmaceutical medicines, especially in elderly patients or patients with chronic illnesses due to their compromised body functions (e.g. renal and hepatic functions in particular). Further research is warranted on the mechanisms of action underlying antidiabetic herb–drug interactions. CYP monoxygenase and P-glycoprotein drug transport pathways are of particular interest given that many antidiabetic medications are subject to metabolism by these enzyme systems [95–97].

It is worth pointing out however, that most studies presented in this review do not distinguish the difference between synergistic and additive effects. A synergistic effect is defined as the total effect produced by a combination of two or more components which is greater than the sum of the individual therapy, whilst an additive effect is simply the sum of individual effects, such that each individual component does not affect the other(s), i.e. no interaction [98]. To this end, it is somewhat problematic to use the term ‘interaction’ unless synergy is proven. Determination of synergism is a complex process especially for HDIs, where numerous bioactive components may be involved. The current models such as isobolographic analysis and the combination index are designed to evaluate the interactions of a small number of active components acting on a single biological target [98]. System-to-system or systems biology methodology is a more appropriate model for the evaluation of more complex interactions but its use is often limited by the availability of the relevant chemical and pharmacological data, especially in complex herbal interventions. Research is essential to develop robust and viable models for assessing herb–drug and herb–herb interactions. Such information is critical to guide the clinical use of these combinations.

There are a number of challenges facing herbal medicine including scant information about their active constituents [99], lack of detailed product information [100, 101], complexity due to multiple chemical components and pharmacological targets [102–104], variation in source of herbal material, lack of standardization and batch–batch reproducibility [105, 106] and of certification of authenticity of herbs used in manufacture [107–109]. Additionally, the existing scientific evidence, particularly clinical, to support the use of herbal medicine remains at the lower levels, and the robustness of the methods used has often been inadequate [110–112]. This highlights the need for further rigorous scientific research to validate the clinical effectiveness and mechanisms of action of herbal medicine as well as complementary medicine in general. Equally important, we need to better our understanding and rigorously document the potential risks associated with herb–drug interactions given the high prevalence of their concurrent use with pharmaceutical medicines, especially for the management of chronic diseases such as diabetes [113–116]. Conversely, it is important to keep in mind that these interactions may also present therapeutic benefits as a result of synergism which may lead to enhanced drug effects or reduced adverse reactions.

In conclusion, interaction between herbal and pharmaceutical agents is a double-edged sword and is of concern to both patients and health care practitioners. It is necessary to continue research on potential risks and benefits associated with these interactions, especially in the cohorts of elderly patients and those who are chronically ill. Such data is critical for the development of future clinical guidelines in order to better health care outcomes.

## Abbreviations

T1DM: type 1 diabetes mellitus
T2DM: type 2 diabetes mellitus
HDI: herb–drug interaction
STZ: streptozotocin
V/F: final velocity
CYP450: cytochrome P450
